# Association between pain phenotype and disease activity in rheumatoid arthritis patients: a non-interventional, longitudinal cohort study

**DOI:** 10.1186/s13075-019-2042-4

**Published:** 2019-11-29

**Authors:** P. M. ten Klooster, N. de Graaf, H. E. Vonkeman

**Affiliations:** 1 Centre for eHealth and Well-being Research, Department of Psychology, Health, and Technology, University of Twente, Enschede, Netherlands; 2 Department of Rheumatology and Clinical Immunology, Medisch Spectrum Twente Hospital, Koningsplein 1, 7512 KZ Enschede, Netherlands

**Keywords:** Rheumatoid arthritis, Pain assessment and management, Patient attitude to health, Outcome measures, Fibromyalgia

## Abstract

**Background:**

In well-controlled rheumatoid arthritis (RA) without significant joint damage, a substantial proportion of patients complain of persistent pain. Previous studies have identified different pain phenotypes in RA, in which non-nociceptive pain phenotypes are associated with higher concurrent disease activity scores. In this longitudinal study, we explored associations between pain phenotypes and long-term disease activity outcome in RA patients. Secondly, we explored whether pain phenotype is associated with comorbid conditions.

**Methods:**

One hundred eighty established RA patients were classified with a nociceptive (61%) or a non-nociceptive (39%) pain phenotype, based on their responses to the painDETECT-questionnaire. Two years of clinical follow-up data on disease activity outcomes were collected. Information on comorbid diseases was derived from electronic patient files.

**Results:**

Patients with a non-nociceptive pain phenotype showed higher mean disease activity scores (DAS28, 2.57; 95% CI, 2.37–2.77 vs. 2.11; 95% CI, 1.94–2.27; *p* < 0.001) and a twofold lower chance of achieving sustained DAS28 remission (OR = 0.49; 95% CI, 0.26–0.92; *p* = 0.020). Only the tender joint count and patient global health significantly differed between the pain phenotype groups. Patients with a non-nociceptive pain phenotype had more often been diagnosed with concurrent fibromyalgia (9.9% vs. 0.9%; *p* = 0.007) and other pain-associated comorbid diseases (52.1% vs. 35.8%; *p* = 0.030) compared with patients with a nociceptive pain phenotype.

**Conclusion:**

This longitudinal study showed consistently worse long-term disease activity outcomes in RA patients with a non-nociceptive pain phenotype which appeared to be mainly due to differences in the subjective components of the disease activity score.

**Trial registration:**

The DREAM cohort study is registered in the Netherlands Trial Register: NTR578.

## Introduction

In recent years, there have been many improvements in the treatment of rheumatoid arthritis (RA), and in most patients, joint inflammation can now generally be well controlled [1]. However, pain often remains problematic in RA [2]. In the absence of joint damage, a substantial number of patients (12–20%) report persistent pain despite having no objective signs of inflammation and low disease activity scores [3]. These findings might be suggestive of a non-inflammatory pain component [4]. This could be very relevant for clinical management of RA, since subgroups of patients with different pain mechanisms may respond differently to treatment [5].

On average, RA patients demonstrate lower pain thresholds and also more signs of hyperalgesia, an increased response to a stimulus which is normally painful, compared with healthy controls [6]. The widespread distribution of hyperalgesia in RA, in the absence of signs of persistent local inflammation or of local destruction of tissues, suggests that the underlying mechanism of persistent pain may originate from central pain regulatory mechanisms, such as loss of conditioned pain modulation or central sensitization, rather than persistent peripheral stimuli of nociceptors [6–8].

The painDETECT questionnaire (PD-Q) was developed in 2006 to identify neuropathic pain components in patients with chronic low back pain [9]. It includes nine questions which address the quality of pain, the pain course pattern, and the radiating feeling of pain. The PD-Q has shown a sensitivity of 85% and a specificity of 80% for detecting neuropathic pain components in patients with low back pain, with the diagnosis by expert pain physicians using all diagnostic methods considered appropriate as gold standard [9]. It has subsequently been recommended as a reliable and applicable screener for pain phenotypes in other chronic disorders as well [10–12]. Increasingly, this questionnaire is being used to identify non-nociceptive pain in RA. For instance, an exploratory study in 159 RA patients by Koop et al. showed that according to the PD-Q screener, almost 40% of the RA patients could be classified as having possible or likely non-nociceptive pain [13]. This was confirmed in a cross-sectional study using the PD-Q by Christensen et al. in 2016, which additionally showed that RA patients who experienced non-nociceptive pain had more tender points and higher 28-joint disease activity scores (DAS28) [14]. However, little is known on the longer-term consequences of nociceptive and non-nociceptive pain patterns on RA disease activity.

Besides the different pain mechanisms, several studies suggest that different comorbid conditions may also independently alter commonly used RA-specific outcome measures, including composite disease activity measures such as the DAS28, categorical states of remission, and functional disability assessments [15]. A recent study by Radner et al. showed that the burden of comorbid disease might have a contributing impact on the patient’s perception of RA disease activity, which in this study was mainly explained by differences in perceived pain and fatigue [16].

The first aim of this longitudinal study was to explore the course of disease activity in RA patients with different pain phenotypes. Secondly, we explored the associations between comorbid diseases and different pain phenotypes. While previous studies examining associations between pain and disease activity in RA have mostly been cross-sectional, we now examined longitudinal differences in DAS28 scores and remission rates between patients with predominantly nociceptive or non-nociceptive pain phenotypes, as determined by the PD-Q screener.

## Materials and methods

### Patients and study design

The study was designed as a non-interventional, longitudinal cohort study, examining data of RA patients participating in the Dutch Rheumatoid Arthritis Monitoring (DREAM-RA) registry. In DREAM-RA, disease activity, patient-reported outcomes, medication, adverse events, laboratory results, and radiographic progression are continually monitored and registered through the web-based data management system *mijnreumacentrum.nl*. The current study is a 2-year longitudinal follow-up study of the cross-sectional study by Koop et al. [13] with an extended inclusion period. Between January 1, 2013 and December 31, 2014, all participating RA patients from the Medisch Spectrum Twente hospital, in Enschede, the Netherlands, were asked to fill out the PD-Q screener in the DREAM registry.

For the current study, all RA patients with a completed PD-Q and with a DAS28 follow-up of at least 2 years were included in the study. Follow-up data on disease activity, remission status, and comorbid diseases were collected from 2013/2014 until censoring on December 31, 2016. In total, data from 180 RA patients was available for analysis. All participants gave online informed consent for this specific sub-study. The DREAM cohort study is registered in the Netherlands Trial Register: NTR578.

### Variables and measures

#### PainDETECT (PD-Q) screener

The total score of the PD-Q ranges between − 1 and 38. A score < 13 indicates the likely presence of nociceptive pain, and a score > 18 indicates a likely neuropathic pain component. With scores between 12 and 19, the result is considered uncertain [9]. As previous studies [13, 14] have shown that there are no substantial differences between RA patients in the medium vs. high PD-Q classification groups, in the current study, PD-Q scores ≥ 13 were interpreted as an indicator of a non-nociceptive pain component.

#### Health Assessment Questionnaire Disability Index (HAQ-DI)

The HAQ-DI is a widely used questionnaire among patients with rheumatic diseases to determine physical function and disability. The HAQ-DI consists of 20 questions in eight different categories of daily life activities (e.g., dressing, eating, walking, hygiene), each resulting in a score between 0 and 3. Scores of 0 to 1 represent mild to moderate disability, 1 to 2 moderate to severe disability, and 2 to 3 severe to very severe disability [17].

#### Short Form 36 Health Survey (SF-36)

The SF-36 is one of the most used tools for measuring health-related quality of life. The questionnaire is based on 36 questions containing eight health concepts. By summarizing these concepts, a mental component summary (MCS) and a physical component summary (PCS) can be created. A higher score indicates a better health-related quality of life [18, 19].

#### Visual analog scale-general health (VAS-GH)

Patients rated their general health on a 0–100 mm VAS with higher scores indicating worse health.

#### Medication use

The use of conventional painkillers (paracetamol, non-steroidal anti-inflammatory drugs (NSAIDs), and/or opioids) and of central nervous system-acting medication (antidepressants and neuromodulators) was self-reported by all participants. Anti-rheumatic drugs were continuously registered by the rheumatologists and rheumatology nurses.

#### Disease activity

In accordance with national and international guidelines [20, 21], disease activity was systematically measured every 3 to 6 months, at each scheduled or unscheduled visit to the outpatient clinic. The DAS28 was developed to measure disease activity in RA and to evaluate the efficacy of treatments in individuals as well as at group level [22]. The DAS28-ESR is calculated with a formula based on the outcomes of erythrocyte sedimentation rate (ESR, mg/l), tender and swollen joint counts (TJC and SJC), and the patient global assessment of general health (VAS-GH) [23–25]. TJC and SJC were performed by the rheumatologist or a trained rheumatology nurse each time the patient visited the clinic. DAS28 scores can be categorized as remission [DAS28 ≤ 2.6], low disease activity [2.6 < DAS28 ≤ 3.2], moderate disease activity [3.2 < DAS28 ≤ 5.1], and high disease activity [DAS28 > 5.1] [26].

#### Comorbid diseases

All patients’ medical files (e.g., referral letters, medical correspondence, and the diagnosis treatment codes (DBC)) were queried to establish the patients’ comorbidities. Comorbid diseases were categorized into nine groups. The EULAR has proposed six main comorbidity groups that are particularly relevant for patients with inflammatory arthritis (cardiovascular diseases, malignancies, infections, gastrointestinal diseases, osteoporosis, and depression) [27]. Three additional comorbidity groups were considered relevant because they could influence the experience of pain and were therefore included in this study: fibromyalgia, pain-associated diseases (e.g., hernia nucleus pulposus, osteoarthritis, and fractures), and remaining miscellaneous comorbidities such as hemangiomas.

### Statistical analyses

The analyses were performed with IBM SPSS Statistics 23. Descriptive statistics were reported as mean with standard deviation when continuous and normally distributed or as median with interquartile range when non-normally distributed. Categorical variables were shown as numbers with percentages. Independent *t* tests or Mann-Whitney tests were used as appropriate to compare the characteristics between patients with and without nociceptive pain for continuous variables. For categorical variables, the chi-square or Fisher exact test was used as appropriate. A two-tailed *p* value < 0.05 was set as the threshold for statistical significance, without correction for multiple testing. Odds ratios (ORs) for the prevalence of comorbid diseases in non-nociceptive versus nociceptive pain phenotype groups were computed using logistic univariate regression analyses.

Linear mixed models (LMMs) with group, time, and time*group interaction as fixed variables were used to analyze and compare the trajectories of DAS28 scores and scores on the individual DAS28 components between the pain phenotypes in the follow-up period. An unstructured repeated covariance matrix was used for all LMMs, as this structure showed the best fit to the data across the disease activity variables.

To compare the long-term outcomes and sustained remission rates between the two pain phenotype groups, univariate logistic or univariate linear regression analyses were used.

## Results

A total of 217 RA patients completed the PD-Q in 2013/2014. Three of the 217 patients were excluded because their diagnosis was later changed to spondyloarthritis. Another 34 were excluded because they either had less than six DAS28 assessments or had less than 2 years of DAS28 follow-up. The excluded patients did not significantly differ in age, gender, and disease duration from the 180 included patients. However, they did have lower mean baseline DAS28 scores (1.86 ± 0.71 vs. 2.39 ± 1.19, *p* = 0.002). The difference was only significantly lower for the TJC component of the DAS28. Of the final 180 patients, 144 (80%) had also been included in the previous study by Koop et al. [13]. Based on the predefined PD-Q cutoff score ≥ 13, 109 (61%) patients were included in the nociceptive pain phenotype group and the remaining 71 (39%) in the non-nociceptive pain phenotype group.

### Patient characteristics

Table 1 displays the baseline characteristics of the nociceptive vs. non-nociceptive pain phenotype groups. Patients in the non-nociceptive pain phenotype group had on average a slightly higher BMI compared with patients in the nociceptive pain phenotype group (*p* = 0.009). Also, the proportion of anti-cyclic citrullinated peptide (anti-CCP)-positive patients was significantly lower in the non-nociceptive pain phenotype group (*p* = 0.022). Significantly more patients in the non-nociceptive pain phenotype group self-reported the use of conventional painkillers (*p* < 0.001). There was no difference in registered use of anti-rheumatic drugs (e.g., disease-modifying anti-rheumatic drugs (DMARDs), NSAIDs) at baseline. Disease duration was comparable between groups.

**Table 1:** Patient baseline characteristics across pain phenotypes

	Nociceptive pain phenotype (*n* = 109)	Non-nociceptive pain phenotype (*n* = 71)	*p*
Female, *n* (%)	67 (61.5)	51 (71.8)	0.153
Age (years), mean (SD)	60.9 (10.46)	60.1 (9.52)	0.617
BMI (kg/m^2^), mean (SD)*	25.4 (4.20)	27.6 (4.87)	0.009
Smoking, *n* (%)	18 (16.5)	15 (21.1)	0.434
Alcohol use, *n* (%)	85 (78.0)	50 (70.4)	0.252
Education, *n* (%)			0.077
Low	38 (34.9)	36 (50.7)	
Middle	47 (43.1)	26 (36.6)	
High	24 (22.0)	9 (12.7)	
Disease duration (years), median (IQR)	8 (5–14)	8 (5–13)	0.644
Anti-CCP positive, *n* (%)**	72 (72.7%)	36 (55.4%)	0.022
RF positive, *n* (%)***	78 (75.7%)	47 (67.1%)	0.216
Self-reported painkillers, *n* (%)			
Conventional painkillers	44 (40.4)	51 (71.8)	< 0.001
Antidepressants/neuromodulators	4 (3.7)	6 (8.5)	0.156
DAS28, mean (SD)	2.13 (1.14)	2.77 (1.19)	0.001
TJC, median (IQR)	1 (0–1)	1 (0–2)	0.007
SJC, median (IQR)	0 (0–1)	0 (0–1)	0.919
ESR, median (IQR)	8 (3–17)	9 (5–17)	0.394
VAS-GH, median (IQR)	10 (3–30)	40 (20–59)	< 0.001
Medication use, *n* (%)			
csDMARD only	62 (56.9)	44 (62.0)	0.497
csDMARD + bDMARD	29 (26.6)	15 (21.1%)	0.403
bDMARD only	11 (10.1)	6 (8.5)	0.713
NSAID only	2 (1.8)	3 (4.2)	0.336
Painkiller only	1 (0.9)	0 (0)	0.420
HAQ-DI (0–3), median (IQR)	0.5 (0–0.88)	1.25 (0.75–1.50)	< 0.001
SF-36 (0–100), mean (SD)			
MCS	52.70 (9.49)	45.24 (10.56)	< 0.001
PCS	43.77 (8.40)	36.45 (8.17)	< 0.001
VAS pain (0–100), median (IQR)	14 (5–30.5)	45.5 (19–62.3)	< 0.001

*BMI* body mass index, *Anti-CCP* anti-cyclic citrullinated peptide, *RF* rheumatoid factor, *DAS28* disease activity score, *TJC* tender joint count, *SJC* swollen joint count, *ESR* erythrocyte sedimentation rate, *VAS* visual analog scale, *DMARD* disease-modifying anti-rheumatic drug, *csDMARD* conventional synthetic DMARD, *bDMARD* biological DMARD, *HAQ-DI* Health Assessment Questionnaire, *SF-36* Short-Form Health Survey, *MCS* mental component summary, *PCS* physical component summery. *BMI was available only in 76 nociceptive patients and 53 non-nociceptive pain patients. **Anti-CCP was available in 99 nociceptive pain patients and 65 non-nociceptive pain patients. ***RF was available in 103 nociceptive pain patients and 70 non-nociceptive pain patients

The mean baseline DAS28 was below the remission cutoff value of 2.6 in the nociceptive pain phenotype group. This was significantly lower than the mean baseline DAS28 in the non-nociceptive pain phenotype group, which was in the range of low disease activity [2.6 > DAS28 ≤ 3.2] (*p* = 0.001). The higher score on the DAS28 in the non-nociceptive pain group was caused by significantly higher tender joint count scores (*p* = 0.007) and worse patient assessment of general health (*p* < 0.001). More objective markers of disease activity (ESR and swollen joint counts) were not significantly different between the groups.

The non-nociceptive pain group additionally reported significantly lower mental and physical quality of life and more disability (*p* < 0.001)*.* Perceived pain intensity was also substantially higher in the non-nociceptive pain phenotype group, with a median VAS score of 45.5 out of 100 compared with 14 out of 100 in the nociceptive pain phenotype group.

### Comorbid diseases

Patients in the non-nociceptive pain phenotype group had slightly more comorbidities compared with the nociceptive pain phenotype group, but this difference was not significant (Table 2). Only fibromyalgia (FM) (clinical diagnosis) and pain-associated comorbid diseases were significantly more common in the non-nociceptive pain phenotype group. Seven patients (10%) in the non-nociceptive pain phenotype group had been diagnosed with comorbid FM, compared with only one patient (1%) in the nociceptive pain phenotype group. Patients with the non-nociceptive pain phenotype had almost a 12-fold increased odds for concomitant FM compared with the nociceptive pain patients (OR = 11.8; 95% CI, 1.42–98.2; *p* = 0.004). Additionally, they had almost a twofold increased risk to have a pain-associated comorbid disease (OR = 1.95; 95% CI, 1.06–3.59; *p* = 0.041).

**Table 2:** Association between pain phenotype and presence of comorbidities

	Nociceptive pain phenotype	Non-nociceptive pain phenotype	OR (95% CI)	*p*
Comorbidity total, mean (SD)	5.92 (3.71)	6.42 (4.10)	1.03 (0.96–1.12)	0.391
Cardiovascular, median (IQR)	0 (0–1)	0 (0–1)	1.08 (0.59–1.98)	0.973
Malignancies, median (IQR)	0 (0–0)	0 (0–0)	0.96 (0.44–2.13)	0.895
Infections, median (IQR)	0 (0–1)	0 (0–1)	0.73 (0.40–1.34)	0.381
Gastrointestinal, median (IQR)	0 (0–1)	0 (0–1)	1.18 (0.61–2.28)	0.614
Pain associated, median (IQR)	2 (0.5–3)	3 (1–4)	1.95 (1.06–3.59)	0.041
Remaining, median (IQR)	1 (0–2)	1 (0–3)	0.64 (0.33–1.22)	0.681
Osteoporosis, *n* (%)	14 (12.8%)	7 (9.9%)	0.74 (0.28–1.94)	0.544
Depression, *n* (%)	4 (3.7%)	0 (0%)	N/A	
Fibromyalgia, n (%)	1 (0.9%)	7 (9.9%)	11.8 (1.42–98.2)	0.004

### Disease activity

**Fig. 1:**
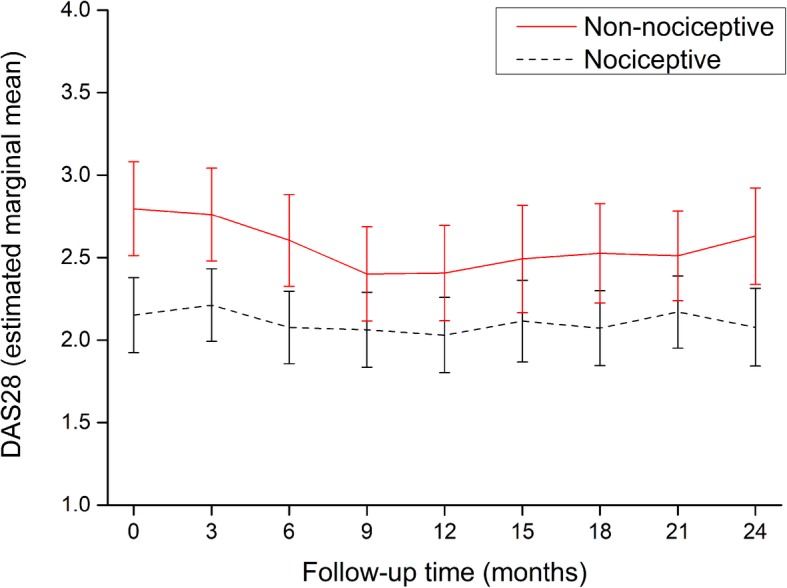
Mean disease activity score in 28 joints over time in nociceptive (black dashed line) and non-nociceptive (red solid line) pain phenotype patients. Error bars are 95% confidence intervals

Figure 1 shows the repeated measures analysis of the DAS28 scores during the follow-up period. The DAS28 had a slightly fluctuating course in both groups. Comparison between the pain phenotype groups showed a significantly higher disease activity score across all time points in the non-nociceptive pain phenotype group. This was confirmed by a significant group effect in the linear mixed model (Table 3). Overall mean values for the DAS28 in the non-nociceptive and nociceptive pain groups over time were 2.57 (95% CI, 2.37–2.77) vs. 2.11 (95% CI, 1.94–2.27), respectively. There was no significant change in DAS28 scores over time for the total group in the follow-up period (*p* for time = 0.128). The time*group interaction was not significant, indicating that the trajectories of the DAS28 did not significantly change over time between groups.

**Table 3:** Results of mixed model analyses of DAS28 and DAS28 component scores over time

	DAS28	TJC	SJC	ESR	VAS-GH
Time	1.60 (*p* = 0.128)	1.11 (*p* = 0.357)	1.65 (*p* = 0.115)	2.26 (*p* = 0.027)	0.71 (*p* = 0.680)
Group	12.15 (*p* < 0.001)	16.99 (*p* < 0.001)	1.78 (*p* = 0.184)	0.19 (*p* = 0.663)	31.17 (*p* < 0.001)
Time*group	0.69 (*p* = 0.697)	0.34 (*p* = 0.950)	1.07 (*p* = 0.385)	1.52 (*p* = 0.157)	0.84 (*p* = 0.573)

Values are *F* values of fixed effects with *p* values in brackets*. DAS28* disease activity score based on 28 joints, *TJC* tender joint count, *SJC* swollen joint count, ESR erythrocyte sedimentation rate, *VAS-GH* visual analog scale for general health

**Fig. 2:**
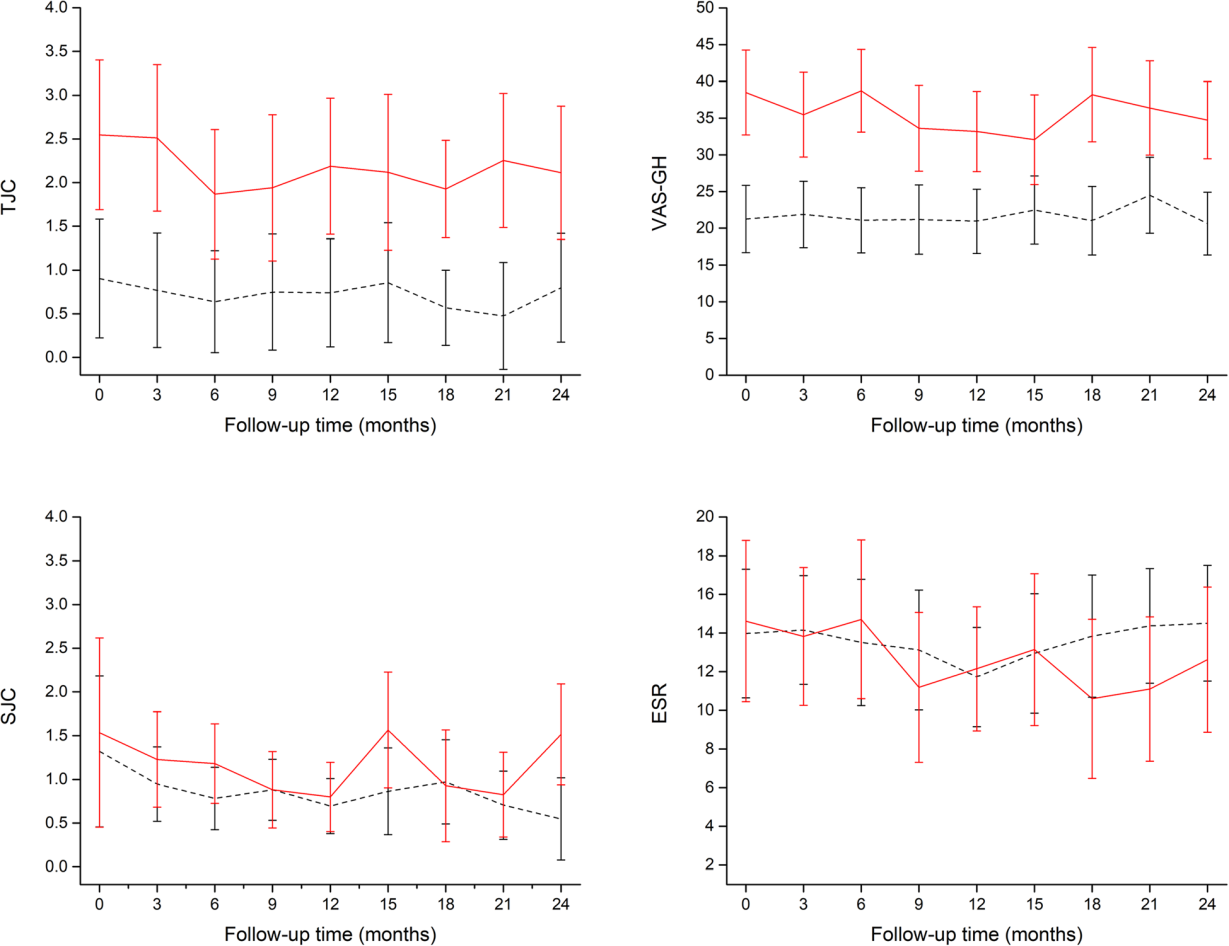
Mean scores of the single components of the disease activity score in 28 joints over time in nociceptive (black dashed line) and non-nociceptive (red solid line) pain phenotype patients. Error bars are 95% confidence intervals. TJC = tender joint count; SJC = swollen joint count; VAS-GH = visual analog scale for general health; ESR = erythrocyte sedimentation rate

Like the baseline scores, only the tender joint count scores and the VAS general health scores were significantly different between the pain phenotype groups over time (Table 3 and Fig. 2). No significant differences in the two more objective components of the DAS28, the swollen joint count, and ESR scores were apparent between pain phenotypes. None of the time*group interactions for the four individual components of the DAS28 were significant.

### Sustained remission

Sustained remission was further categorized into periods of short (between 6 and 9 months), medium (between 9 and 12 months), and long (12 months or longer) sustained remission.

There was a large discrepancy in achieving sustained remission across the two pain phenotype groups. A total of 79/109 (72.5%) of the patients in the nociceptive pain phenotype group achieved a sustained remission of 6 months at least once during follow-up (Table 4). In the non-nociceptive pain group, a significantly lower proportion of only 39/71 (54.9%) achieved sustained remission. Patients in the non-nociceptive pain phenotype group had a twofold decreased chance of achieving sustained remission (OR = 0.49; 95% CI, 0.26–0.92; *p* = 0.02) and an even smaller chance of achieving remission for at least 12 months (OR = 0.42; 95% CI, 0.23–0.78; *p* = 0.006). A medium and long sustained remission of 9 and 12 months, respectively, was achieved by 26/71 (36.6%) and 32/71 (45.1%) of the non-nociceptive pain patients, compared with 67/109 (61.5%) and 63/109 (57.8%) of the nociceptive pain patients.

**Table 4:** Disease activity and DAS28 scores and remission rates in the 2-year follow-up period

	Nociceptive pain phenotype	Non-nociceptive pain phenotype	OR (95% CI)	*p*
DAS28, mean (SD)	2.14 (0.85)	2.55 (0.87)	1.74 (1.21–2.50)	0.002
DAS28 categories, *n* (%)			1.44 (0.76–2.72)	0.062
Remission/low disease activity	95 (88.0)	55 (77.5)		
Moderate/high disease activity	13 (11.9)	16 (22.5)		
Remission achieved, *n* (%)				
SSR	79 (72.5)	39 (54.9)	0.49 (0.26–0.92)	0.020
MSR	67 (61.5)	32 (45.1)	0.51 (0.28–0.94)	0.032
LSR	63 (57.8)	26 (36.6)	0.42 (0.23–0.78)	0.006

*SSR* short sustained remission (6–9 months), *MSR* medium sustained remission (9–12 months), *LSR* long sustained remission (≥12 months)

## Discussion

Rheumatoid arthritis has always been considered an inflammatory joint disease, causing inflammatory or nociceptive pain. In this study, however, only 61% of the RA patients fulfilled the criteria for a nociceptive pain phenotype, as categorized by the PD-Q. A previous study also using the PD-Q in RA by Christensen et al. in 2016 showed comparable proportions of nociceptive and non-nociceptive pain phenotypes: 65% of the RA patients experienced nociceptive pain and the remaining 35% non-nociceptive pain [14]. These results suggest that pain in RA cannot be not be fully explained by nociceptive pain mechanisms alone. Many studies have now identified alternative pain mechanisms; neuropathic pain or central sensitization may also play a significant role in RA [28–31]. Research has shown that in RA, tenderness and pain may also be present in apparently healthy articular and non-articular tissues [31]. Furthermore, there are frequent indications for the occurrence of allodynia and hyperalgesia in RA, also in the absence of local inflammation or of local destruction of tissues [28–31]. The occurrence of allodynia and hyperalgesia suggests that there could be an amplification of the transmission of nociceptive information, which may be indicative of pain sensitization in RA patients [31].

The lack of a golden standard to measure the relatively new concept of central sensitization is a common problem in pain research. Furthermore, features that may be indicative of central sensitization tend to overlap with features indicative of neuropathic pain, making it difficult to distinguish between the two [32]. Therefore, in our current study, we focused on merely differentiating between nociceptive pain and non-nociceptive pain, based on screening for pain characteristics using the PD-Q.

In the current study, RA patients with a non-nociceptive pain phenotype were found to have a nearly 12-fold increased risk to have a concurrent clinical diagnosis of fibromyalgia. This is however not unexpected. One of the theories on the pathophysiology of fibromyalgia (FM) is based around the concept of central sensitization; therefore, the American College of Rheumatology (ACR) fibromyalgia diagnostic criteria are commonly used to define central sensitization [33]. Assuming that RA patients with a non-nociceptive pain phenotype could be suffering from central sensitization, it would not be surprising that nearly all the RA + FM patients in this cohort demonstrated a non-nociceptive pain phenotype.

Patients in the current non-nociceptive pain phenotype group had more severe rheumatic disease and worse treatment outcomes, i.e., higher mean disease activity scores and lower remission rates. This has also commonly been reported in RA patients with concurrent FM [34]. Moreover, other studies of patients with a predominantly central sensitization origin of pain also report more severe pain and lower scores on health-related quality of life domains [35]. A previous cross-sectional study by Lee et al. showed that in RA patients, elevations in disease activity measures were associated with pain sensitization [36]. A similar finding was observed in our previous cross-sectional study, which showed that patients with non-nociceptive pain had significantly higher tender joint scores and that total DAS28 scores also tended to be higher in this group. The current longitudinal follow-up study extends these findings by showing that the mean DAS28 in RA patients with a non-nociceptive pain phenotype remained consistently higher over the 2-year follow-up than in patients with a nociceptive pain phenotype. Moreover, the current study demonstrated that, both at baseline and during follow-up, these higher DAS28 scores were mostly the result of the subjective components of the DAS28: higher TJC and a worse patient assessment of global health. These findings support the theory that mechanisms other than local inflammation or local tissue destruction could cause increased and persistent pain (or sensitization) in RA and have adverse effects on treatment outcomes.

Interestingly, patients with non-nociceptive pain tended to have a slightly higher BMI and were more often anti-CCP negative. The higher BMI in the non-nociceptive pain phenotype may be related to previous findings showing higher pain severity scores in (especially female) RA patients who are overweight or obese [37, 38]. The mechanism of this association however remains unknown [37]. The lower proportion of anti-CCP-positive patients in the non-nociceptive pain group deserves further study, as a previous study [39] also showed a significant difference in anti-CCP, while both the current study and previous studies have not shown clear differences in RF positivity between pain phenotypes [14].

Pain sensitization is often measured with the PD-Q. However, in the previously mentioned study by Lee et al., sensitization was determined with quantitative sensory testing (QST), including testing for pressure pain thresholds, conditioned pain modulation, and temporal summation, and disease activity was measured with the Clinical Disease Activity Index (CDAI), a composite measure that includes a TJC, SJC, patient global assessment, and assessor global assessment on disease activity. The study by Lee et al. corroborates our current results on disease activity in RA and especially on the more subjective components of the disease activity scores: the TJC and patient global assessment of disease activity were also significantly higher in patients with indications of pain sensitization, while the SJC did not differ between the groups [36]. The SJC and ESR are arguably more objectively related to inflammation, being evaluator-observed and laboratory measures. In clinical and research settings, attention should be paid to both the individual components as well as the sum scores of composite measures of RA disease activity. The current DAS28 includes pain and global health assessments which may not be entirely dependent on the inflammatory pathways of the disease or on the disease activity itself.

Although there was a significant difference in mean DAS28 scores between the two pain phenotype groups, on average both were below the remission cutoff value of 2.6 during the follow-up period. However, to achieve “sustained” remission, the disease activity score must be below 2.6 at every assessment over a period of at least 6 months. In the nociceptive pain group, 72% of the patients reached sustained remission compared with 55% of the non-nociceptive pain patients. Similar findings were found for achieving 9 or 12 months of sustained remission. These results are congruent with previous research, because known predictive factors for reaching sustained remission are earlier time to remission, lower baseline disease activity, mild disability, lower TJC, and less pain [40, 41]. The nociceptive pain patients in the current study met all of these features (only time to remission was unknown) and had notably higher chances of achieving six or more months of sustained remission.

A remarkable finding in the current study was the comparable use of anti-rheumatic drugs in both groups. Although the nociceptive pain group used slightly more biological DMARDs than the non-nociceptive pain group, this difference was not significant. The lack of difference may be explained by the mean disease activity scores. In both groups, the mean DAS28 was below 3.2, which indicates low disease activity [26]. Although there is a significant difference in disease activity scores between the pain phenotypes, a score below 3.2 may not be clinically relevant enough to drive a different treatment approach. Furthermore, it is possible that the physicians base their motivation whether or not to change doses or to switch types of anti-rheumatic drugs mainly on the more objective markers like the SJC and ESR, rather than on the more subjective TJC or the patient’s assessment of global health. A previous Dutch study in 2011 examined factors that influence decisions to escalate treatment in RA. For rheumatologists, the most influential factors were the physician global assessment of disease activity, the SJC, and the comparison between disease activity now and 3 months ago [42], which are all more or less objective markers of disease activity.

To our knowledge, this is the first longitudinal study examining associations between different pain phenotypes and long-term disease activity outcomes in RA patients. A previous cross-sectional study by Koop et al. demonstrated the presence of different pain phenotypes in RA patients [13]. Our current study showed that these different pain phenotypes influence long-term treatment outcomes. RA treatment is mainly anti-inflammatory and targets the nociceptive pain pathway. Failure to recognize and adequately treat different underlying pain mechanisms could negatively impact outcomes that matter to the patient. Possibly, predetermination of a patient’s pain phenotype before starting treatment may prevent overtreatment with anti-inflammatory drugs and lead to better treatment outcomes through personalized treatment of pain.

## Conclusions

This longitudinal study of 2-year follow-up showed that RA patients with a non-nociceptive pain phenotype reported higher pain scores, more severe disability, and a lower physical and mental quality of life compared with RA patients with a nociceptive pain phenotype. Concomitant fibromyalgia was more common in patients with a non-nociceptive pain phenotype. Furthermore, patients with a non-nociceptive pain phenotype showed consistently worse disease activity outcomes, with higher mean DAS28 scores and lower rates of sustained remission. Both at baseline and during follow-up, these higher disease activity scores were mostly caused by the more subjective components of the DAS28: a higher TJC and a worse patient assessment of global health.

## Data Availability

Please contact the author for data requests.

## Abbreviations

ACR: American College of Rheumatology
CDAI: Clinical Disease Activity Index
CI: Confidence interval
DAS28: 28-Joint disease activity score
DBC: Diagnosis treatment codes
DMARD: Disease-modifying anti-rheumatic drug
DREAM-RA: Dutch Rheumatoid Arthritis Monitoring registry
ESR: Erythrocyte sedimentation rate
EULAR: European League Against Rheumatism
FM: Fibromyalgia
HAQ-DI: Health Assessment Questionnaire Disability Index
LMM: Linear mixed models
NSAID: Non-steroidal anti-inflammatory drug
OR: Odds ratio
PD-Q: PainDETECT questionnaire
QST: Quantitative sensory testing
RA: Rheumatoid arthritis
SF-36: Short Form 36 Health Survey
TJC and SJC: Tender and swollen joint counts
VAS-GH: Visual analog scale-general health
